# Serum micro-rna profiles in patients with autosomal dominant polycystic kidney disease according to hypertension and renal function

**DOI:** 10.1186/s12882-017-0600-z

**Published:** 2017-05-30

**Authors:** Ismail Kocyigit, Serpil Taheri, Elif Funda Sener, Eray Eroglu, Fahir Ozturk, Aydin Unal, Kezban Korkmaz, Gokmen Zararsiz, Murat Hayri Sipahioglu, Yusuf Ozkul, Bulent Tokgoz, Oktay Oymak, Tevfik Ecder, Jonas Axelsson

**Affiliations:** 1 Department of Internal Medicine, Division of Nephrology, Erciyes University Medical Faculty, Kayseri, Turkey; 2 Department of Medical Biology, Erciyes University Medical Faculty, Kayseri, Turkey; 3 Department of Internal Medicine, Erciyes University Medical Faculty, Kayseri, Turkey; 4 Betul-Ziya Eren Genome and Stem Cell Center, Erciyes University Medical Faculty, Kayseri, Turkey; 5 Department of Biostatistics, Erciyes University, Kayseri, Turkey; 6 Department of Internal Medicine, Division of Nephrology, Istanbul Bilim University, Istanbul, Turkey; 7 Division of Vascular Biology, Department of Medical Biochemistry and Biophysics, Karolinska institutet, Karolinska University Hospital, Stockholm, Sweden; 8 Department of Clinical Immunology, Karolinska University Hospital, C2:66 ImmTrans, 141 86 Stockholm, Sweden

## Abstract

**Background:**

Autosomal dominant polycystic kidney disease (ADPKD) is a common hereditary disorder with unclear disease mechanism. Currently, overt hypertension and increased renal volume are the best predictors of renal function. In this study, we assessed the usefulness of selected circulating microRNAs (miRs) to predict disease progress in a cohort with ADPKD.

**Methods:**

Eighty ADPKD patients (44.6 ± 12.7 years, 40% female, 65% hypertensive) and 50 healthy subjects (HS; 45.4 ± 12.7, 44% female) were enrolled in the study. Serum levels of 384 miRs were determined by Biomark Real Time PCR. Groups were compared using the limma method with multiple-testing correction as proposed by Smyth (corrected *p* < 0.01 considered significant).

**Results:**

Comparing ADPKD to HS, we found significant differences in blood levels of 18 miRs (3 more and 15 less abundant). Of these, miR-3907, miR-92a-3p, miR-25-3p and miR-21-5p all rose while miR-1587 and miR-3911 decreased as renal function declined in both cross-sectional and longitudinal analysis. Using ROC analysis, an increased baseline miR-3907 in the circulation predicted a > 10% loss of GFR over the following 12 months (cut-off >2.2 AU, sensitivity 83%, specificity 78%, area 0.872 [95% CI: 0.790–0.953, *p* < 0.001]). Adjusting for age and starting CKD stage using multiple binary logistic regression analysis did not abrogate the predictive value.

**Conclusion:**

Increased copy numbers of miR-3907 in the circulation may predict ADPKD progression and suggest pathophysiological pathways worthy of further study.

**Electronic supplementary material:**

The online version of this article (doi:10.1186/s12882-017-0600-z) contains supplementary material, which is available to authorized users.

## Background

Autosomal dominant polycystic kidney disease (ADPKD) is a congenital progressive disease resulting in chronic kidney disease (CKD) that often progresses to end-stage renal disease (ESRD). In most patients, renal function is sufficient to live a normal life until the fourth to sixth decades of life but thereafter declines [1]. The main predictors of future renal function are the presence of hypertension, increasing kidney volume and decreased renal blood flow [2]. Of these hypertension often significantly predates the development of chronic kidney disease (CKD) [1], while imaging studies to detect the size and amount of remaining parenchyma of the kidneys are resource consuming and not very sensitive [1, 3]. Identifying patients at risk has obvious clinical application and the identification of relevant biomarkers associated with ADPKD CKD-development may also help to elucidate the currently obscure mechanisms linking commonly encountered mutations in the PKD1 and 2 genes to renal cysts and CKD [1].

MicroRNAs (miRs) are small, non-coding RNA molecules that modulate many different intracellular pathways negatively regulating gene expression at the post-transcriptional level. As each miR has multiple putative targets, and as the distribution of miRs vary between cell types and over time, they are likely to play a physiological role [4]. In renal tissue miRs have been implicated in both embryonic development and kidney disease processes and altered levels have been reported in the blood and urine during several specific glomerular and tubular diseases as well as in animal models of these [5–8]. While few studies have investigated miRs in ADPKD, two recent animal experiments have suggested that they may be important as regulators of pro-fibrotic calcium signaling [9, 10].

In the present pilot study, we asked if circulating miRs would differentiate ADPKD patients from healthy subjects under clinically relevant conditions, and if levels of any circulating miR would predict the furure clinical course of ADPKD during one year of follow-up.

## Methods

### Study design and patient selection

Among the patients with ADPKD included in the Turkish Society of Nephrology’s Polycystic Kidney Disease Working Group Registry, we screened those scheduled to receive followed-up by the Medical Faculty of Erciyes University between June 2013 and January 2015. The study was approved by the Erciyes University Ethical Review Board (Kayseri, Turkey) prior to start, and all participants signed written informed consent forms after being informed about the study and given time to consider their participation.

Eligible patients were those aged 18–70 years under regular follow-up and meeting the above diagnostic criteria for ADPKD. Patients with a known glomerular filtration rate (eGFR) <15 ml/min/1.73 m^2^ and those with diagnosed cardiovascular disorders, diabetes mellitus or an active infection were all excluded. The patients were invited to enroll by post, and those that accepted all signed written informed consent forms. Demographic characteristics (e.g. sex, age, education and smoking history), renal manifestations (e.g. hematuria, urinary system infection, urinary tract stones and renal replacement therapy) and cardiovascular manifestations (e.g. hypertension and mitral valve prolapse) were hen recorded using a web-based data collection form. Using the above criteria, 80 ADPKD patients were included in the study. A total of 50 healthy subjects were also recruited by advertisement and gave written, informed consent to participate. Both patients and controls were asked to undergo ambulatory blood pressure monitoring to diagnose the presence or absence of hypertension. Data of 24 h urine samples was collected at inclusion and in the patients again after exactly 12 months of follow-up (*n* = 80) to determine eGFR (mean of urinary creatinine and urea clearances) and proteinuria levels.

### Continuous blood-pressure monitoring

Twenty-four-hour blood pressure monitoring was performed using a Del Mar Medical Ressurometer Model P6 (Del Mar Reynolds, Irvine, CA, USA), and the results were assessed using the companion software. Ambulatory measurements were conducted once every 15 min from 7 a.m. until 11 p.m., and once every 30 min from 11 p.m. until 7 a.m. Evaluation was performed taking the mean values of day and night blood pressures into account. Hypertension was considered to be present if the average systolic pressure was ≥130 mmHg and/or the average diastolic pressure was ≥80 mmHg over the 24-h, or if the individual was taking antihypertensive medication (*n* = 10).

### RNA isolation

Five milliliters of venous blood was drawn and after coagulation centrifuged at 4000 g for 15 min to isolate serum, which was transferred into clean microcentrifuge tubes and again centrifuged (12,000 g, 5 min) with the resulting serum aliquoted into 200 μl and stored at −80 °C until RNA isolation. Total RNA was isolated using Trizol (500 μL) (Roche, Mannheim, Germany) according to the manufacturer’s instructions and stored at -80 °C.

### Reverse transcription (RT) and quantitative PCR

Isolated RNA samples were reverse-transcribed into complementary DNA (cDNA) in 5 μl final reaction volumes using MicroRNA Reverse Transcription Kit (miScript II RT Kit, Cat No: 218,161, Qiagen, Germany). cDNA samples were kept at -80 °C until PCR analysis. Next, we performed pre-amplification using the PreAmp Master Mix (miScript Microfluidics PreAMP Kit, Cat No: 331,455, Qiagen) together with the miScript miRNA PCR Array Human Serum & Plasma 384HC (Cat.No: MIHS-3106Z, Qiagen, Germany) (Additional file 1). For pre-amplification 2 μl 1/5 diluted RT product were added to 3 μl of the PreAmp mix. PreAmp Thermal protocol were as follows: 95 °C for 15 min, followed 94 °C for 120, 55 °C for 60 s and 70 °C for 60 s by 2 cycles, followed by 10 cycles with 94 °C for 30 s, 60 °C for 3 min, finally 99.9 °C for 600 s, and a rest period at 4 °C.

Next, qPCR was performed using the high-throughput BioMark Real-Time PCR system (Fluidigm, South San Francisco, CA, USA). Preamplified cDNA samples were diluted with low EDTA (0.1 mM) TE Buffer (1:5). A BioMark IFC controller HX (Fluidigm, South San Francisco, CA, USA) was used to mix a total of 3.15 μl diluted preAmplified cDNA with appropriate amounts of Universal PCR Master Mix, (miScript Microfluidics PCR Kit, Cat No: 331,431, Qiagen) and 20X GE Sample Loading Reagent (Fluidigm, South San Francisco, CA, USA) in a Dynamic 96.96 array plate microfluidic system (Fluidigm,South San Francisco, CA, USA). Real-Time PCR was performed automatically using a thermal mixing protocol followed by 50 °C for 120 s, 70 °C for 1800 s, 25 °C for 600 s, then followed 95 °C for 600 s. Finally, 23 cycles with 94 °C for 15 s, 55 °C for 30 s, 70 °C for 30 s followed by 60 °C for 60 s. All of the genetic studies were performed by experienced operators in the Genome and Stem Cell Center at Erciyes University (GENKOK).

### Data collection and statistical analyses

Data were collected with the Fluidigm® Real-Time PCR analysis software using the linear baseline correction method and the auto global Cq threshold. System given Cq values of 999 and values larger than 23 haploid genome equivalents (HGEs) were considered as unreliable and removed. Median limit of detection (LOD) Cq values were calculated across all arrays to impute missing values. Data normalization was performed by using the 2^-ΔΔCT^ method. To detect the differentially expressed miRs, the linear models for microarray and RNA-Seq data (limma) procedure was used along with false discovery rate correction of *p* values according to Smyth [11]. Adjusted *p* values less than 0.01 were considered statistically significant.

The DIANA-mirPath software v. 3.0 was used to identify signaling pathways associated with differentially expressed miRs [12] with highly conserved and experimentally verified TarBase v. 7.0 miRs were considered as potential targets and the agglomerative hierarchical clustering algorithm was applied to determine miR and pathway clusters based on their interaction levels. Analyses were conducted using the Rcmdr and limma packages of R v. 3.1.2 (R Core Team, 2014).

To assess the performance metrics of miRs associated with changes in eGFR, ROC curve analysis was conducted. Moreover, univariate and multiple binary logistic regression models were constructed. Age, gender, hypertension, CKD and miR-3907 were included forced into an initial model which was used to identified the best independent subset using forward elimination with maximum likelihood ratio. Odds ratios are given with 95% confidence intervals for each factor in each model.

## Results

### Cohort demographics

Age and gender distributions were similar in the control and patient groups (Table 1). The mean patient eGFR of 74.0 ± 35.4 ml/min/1.73 m^2^ was significantly lower than the 98.1 ± 15.3 ml/min/1.73 m^2^ mean value in control subjects (*p* < 0.05). According to the CKD classification, 30 ADPKD were stage 1 (GFR ≥90 mL/min/ 1.73 m^2^ but albuminuria or hematuria), 22 in stage 2 (60–89 mL/min/ 1.73 m^2^), 20 in stage 3 (30–59 mL/min/ 1.73 m^2^), and 8 in stage 4 (15–29 mL/min/ 1.73 m^2^). None of the healthy subjects had proteinuria or CKD.

**Table 1:** Comparison of demographical and biochemical data between ADPKD patients (*n* = 80) and healthy controls (*n* = 50)

Clinical Parameters	ADPKD patients (*n* = 80)	Healthy controls (*n* = 50)	*p*
Age, year	44.6 ± 12.7	45.4 ± 12.7	0.35
Gender, F/M	33/47	22/28	0.12
Hemoglobin, g/L	13.9 ± 2.1	13.3 ± 1.9	0.74
BMI (kg/m^2^)	27.6 ± 5.37	26.74 ± 7.8	0.21
Average 24-h systolic BP, mmHg	139 ± 7.2	119 ± 6.1	**0.005**
Average 24-h diastolic BP, mmHg	86 ± 4.8	73 ± 3.9	**0.01**
eGFR^a^, mL/min per 1.73 m^2^	74.0 ± 35.4	98.1 ± 15.3	**<0.001**
Smoking, *n*(%)	18 (22)	10 (20)	0.54
Fasting Total cholesterol, mg/dL	197 ± 39	184 ± 33	0.19
HDL- cholesterol, mg/dL	42 ± 10	46 ± 8	0.23
LDL- cholesterol, mg/dL	124 ± 30	119 ± 36	0.63
Proteinuria, g/day	0.47 (0.12–1.19)	0.15 (0.07–0.6)	**<0.001**
Hs-CRP, mg/L	6.9 ± 2.7	3.5 ± 1.6	**0.01**
Albumin (g/dL)	3.9 ± 0.61	4.1 ± 0.62	0.72

*ADPKD* Autosomal-dominant polycystic kidney disease, *eGFR* estimated glomerular filtration rate, *F*/*M* Female/Male, *HDL* High density lipoprotein, *Hs-CRP* High sensitive C-reactive protein, *LDL* Low density lipoprotein

^a^Calculated by the CKD-EPI formula

Data are expressed as mean ± SD or median for normally distributed data and percentage (%) for categorical variables.

Bold values indicate the significant values (*p* < 0.05)

Comparing hypertensive (HT+) to non-hypertensive (HT-) ADPKD patients only (Table 2; based on 24-h blood pressure data), HT+ were older (47.9 ± 11.4 vs. 32.7 ± 8.8 years) and had a lower GFR (58.5 [21–91] vs. 108 [85–116] mL/min/ 1.73 m^2^) than did HT-. Baseline proteinuria did not differ between these groups, nor did the mean proteinuria of the whole patient cohort change longitudinally during follow-up (baseline 0.47 (0.12–1.19) vs 0.54 (0.15–1.32) g/L at 1 year).

**Table 2:** Comparison of demographical and laboratory features between the 28 ADPKD patients without (HT-) and the 52 patients with (HT+) hypertension (defined as average systolic pressure was ≥130 mmHg and/or the average diastolic pressure was ≥80 mmHg during 24-h, or if the individual was taking antihypertensive medication [*n* = 10])

Variables	ADPKD patients (*n* = 80)		*p*
	Non-hypertensives (*n* = 28)	Hypertensives (*n* = 52)	
Age (years)	32.7 ± 8.8	47.9 ± 11.4	**<0.001**
Gender (male)	15 (53.0)	32 (61)	0.07
Hemoglobin, g/l	13.4 ± 1.5	14.0 ± 1.7	0.22
BMI (kg/m^2^)	26.6 ± 5.37	29.74 ± 7.8	0.109
Average 24-h systolic BP, mmHg	124 ± 4.6	150 ± 9.1	**<0.001**
Average 24-h diastolic BP, mmHg	73 ± 3.9	98 ± 7.4	**<0.001**
Smoking status, *n*(%)	6 (21)	12 (23)	0.701
eGFR^a^ ml/min/1.73 m^2^	108 (85–116)	58.5 (21–91)	**<0.001**
Fasting total cholesterol, mg/dl	178.7 ± 41.7	201.79 ± 33.00	**0.02**
Fasting LDL-C, mg/dl	113.3 ± 40.4	124.3 ± 30.6	0.17
Proteinuria, g/day	0.20(0.12–0.33)	0.70(0.4–1.19)	**<0.001**
Albumin (g/dL)	4.0 ± 0.61	3.9 ± 0.54	0.66

*ADPKD* Autosomal-dominant polycystic kidney disease, *BMI* Body mass index, *eGFR* estimated glomerular filtration rate, *LDL* Low density lipoprotein

Values are expressed as *n*(%), mean ± SD or median(1st-3rd quartiles).

^a^Calculated by the CKD-EPI formula

Bold values indicate the significant values (*p*<0.05)

### miR levels in the venous blood of patients and healthy controls

**Fig. 1:**
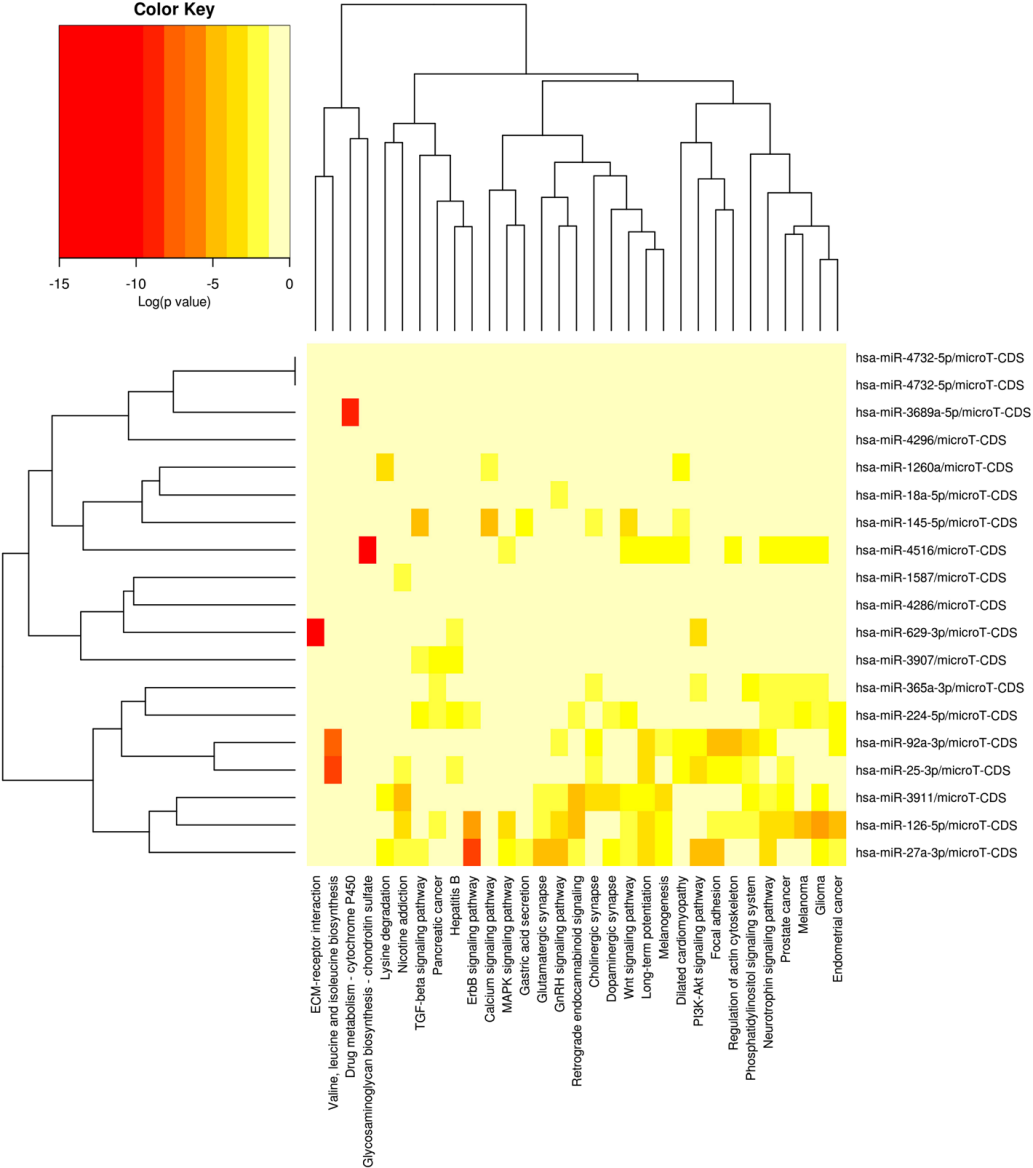
Heatmap-plot of FDR-corrected log(*p*-values) for differences in venous blood miRs between 80 ADPKD patients and 50 healthy controls grouped by gene ontology group (GO). Generated using DIANA-miRPath v. 3.0 (40)

**Fig. 2:**
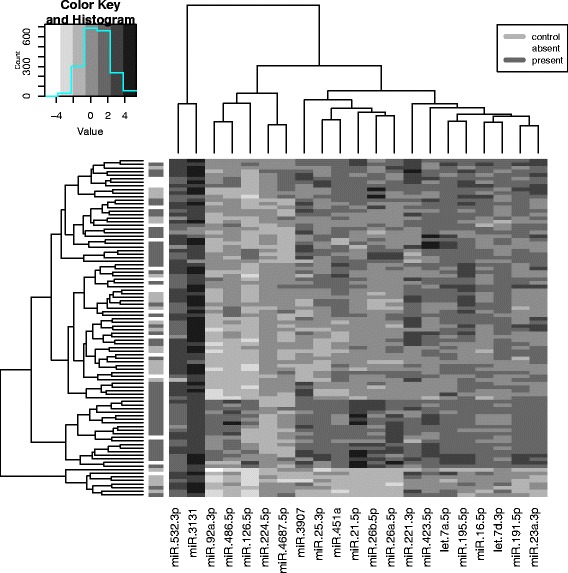
Heatmap-plot displaying clustering of miRNA levels across study participants expressed as fold-change between all patients and all controls. Along the top is a dendrogram showing similarities between miRNAs, while the dendrogram along the left side shows similarities between groups (28 non-hypertensive and 52 hypertensive ADPKD patients and 50 non-hypertensive healthy controls)

Comparing measured whole blood miR levels copy numbersof 18 miRs differed (corrected *p* < 0.01 considered significant; −1.1 to 3.3 fold difference; Figs. 1 and 2). Of these, miR-25-3p, miR-92a-3p and miR-3907 were more abundant while miR-18a-5p, miR-27a-3p, miR-128-3p, miR-145-5p, miR-202-3p, miR-224-5p, miR-365a-3p, miR-629-3p, miR-1587, miR-3689-5p, miR-3911, miR-4286, miR-4296, miR-4516 and miR-4732-5p were all less abundant (Fig. 2; *p* < 0.01 for all).

### miR levels in ADPKD patients with our without hypertension

**Fig. 3:**
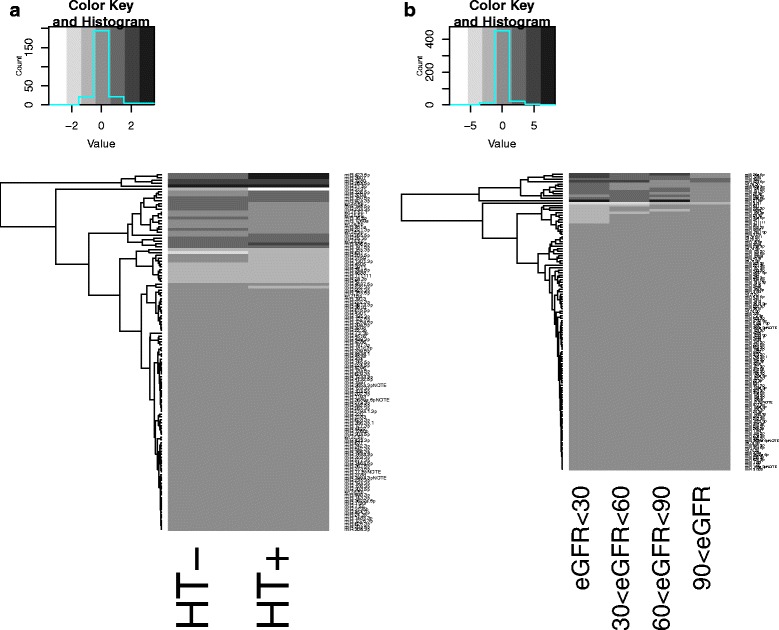
Comparisons of measured differences in whole blood miR levels between **a** ADPKD patients with (HT+; *n* = 52) or without (HT-; *n* = 28) hypertension, and **b** ADPKD patients with CKD stage 1, 2, 3 or 4

Based on 24-h continous blood-pressure monitoring, patients were grouped into hypertensive (HT+; average SBP ≥ 130 mmHg and/or average DBP ≥ 80 mmHg or taking antihypertensive medication [*n* = 10]) or non-hypertensive (HT-). Comparing miRNAs copy numbers between these groups, patients with hypertension had signficantly different levels of 21 miRs when compared to non-hypertensives (Fig. 3A; −1.5 to 2.8 fold difference). Of these, miR-21-5p, miR-25-3p, miR- 26a-5p, miR-26b-5p, miR-92a-3p, miR-126-5p, miR-191-5p, miR-224-5p, miR-423-5p, miR-486-5p, miR-532-3p, miR-3907 and miR4687-5p were more abundant while let-7a-5p, let-7d-3p, miR-16-5p, miR-23a-3p, miR-195-5p, miR-221-3p, miR-451a, miR-3131 were less abundant (corrected *p* < 0.01 for all). However, these differences were not obvious when HT+ and HT- patients were compared with controls (Fig. 3a).

### miR levels in ADPKD patients according to CKD stage

**Fig. 4:**
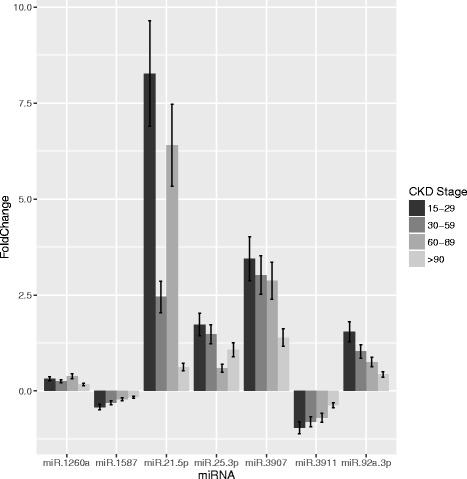
Comparison of circulating levels of selected miRs between patients with various stages of CKD. Adjusted *p*-values computed based on Benjamini-Hochberg correction are reported

When grouped according to CKD stage, we found 8 miRs that showed a dose-response behavior with incremental changes at least twice as GFR declined. These were miR-1260a, miR-1587, miR-21-5p, miR-3907, miR-3911 and miR-92a-3p (**Figs.** **3b**, **4**). Of these, there was a consitent negative linear relationship between eGFR and blood copy numbers for miR-92a-3b and miR-3907 and a positive linear relationship between eGFR and blood copy numbers for miR-3911 and miR-1587. The remaining miRNAs showed a non-linear pattern.

### Patients’ renal function after 1 year and the predictive value of baseline blood miRNAs

As expected, there was a small but statistically significant decrease in eGFR (74 ± 35 to 69 ± 32 mL/min/1.73m^2^; *p* < 0.001) during follow-up. Using data of baseline and follow-up eGFR, we estimated each patients’ decline during the year (median − 5 [range + 2 to −29] mL/min/1.73m^2^/ year). Patients were then grouped into two groups according to if their loss over the period was ≤10% or >10% of their baseline value. 30 patients had progression rate above 10%.

**Fig. 5:**
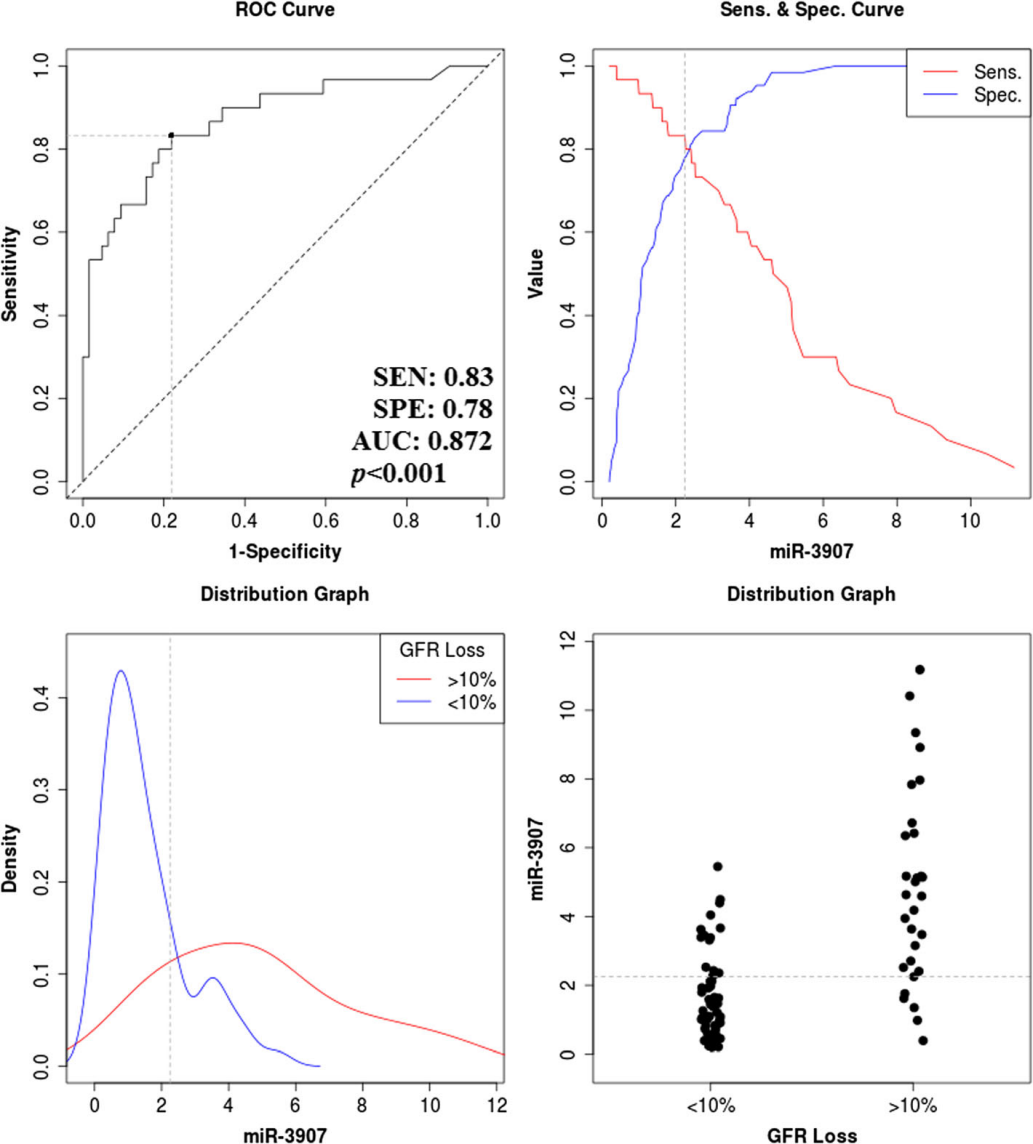
ROC analysis of baseline blood levels of miR-3907 as a predictor of progressive loss of eGFR > or ≤10% during 12 months of follow up in 80 ADPKD patients. **a** ROC curve and the identified cut-off value of miR-3907. **b** Sensitivity and specificity values around the cut-off value. **c** Density plots showing the distribution of patients who have GFR loss larger and smaller than 10%. **d** Scatter diagram of patients around the cut-off value

Next, we assessed the relative predictive value of the miRs that differed significantly between hypertensive and non-hypertensive ADPKD to predict the future loss of eGFR >10%. Of these, only miR-3907 was significantly predictive in ROC analysis (Fig. 5). The sensitivity and specificity of miR-3907 were 83% and 78% (cut-off >2.2 AU) with an area of under the ROC curve of 0.872 (95% CI: 0.790–0.953, *p* < .0001).

Additionally, we performed univariate and multiple binary logistic regression analysis comparing the realtionship between selected variables and progression outcomes in all patients. A loss of eGFR >10% during follow-up was independently associated with age, CKD stage and miR-3907 (Table 3).

**Table 3:** Univariate and multiple binary logistic regression models investigating the robustness of baseline blood miR-3907 as a predictor of GFR loss during 12 months of follow-up in 80 ADPKD patients. A decrease of >10% of baseline eGFR at follow-up was defined as progression

Variable	Univariate		Multivariate			
	Progression OR (95% CI)	*p*	Initial forward logistic regression model		Final logistic regression model	
			Progression OR(95% CI)	*p*	Progression OR(95% CI)	*p*
**Age** *(above* vs. *below median)*	1.11(1.05–1.18)	**<0.001**	1.30(1.07–1.58)	**0.008**	1.28(1.09–1.51)	**0.003**
**Gender** *(if female)*	1.47(0.62–3.51)		3.59(0.62–20.89)			
**Hypertension** *(if present)*	9.58(2.64–34.79)	**<0.001**	0.75(0.03–19.49)	ns		
**CKD stage**						
4	1.00	**<0.001**	1.00	**0.032**	1.00	**0.014**
3 *(*vs. *stage 4)*	1.35(0.26–7.07)	ns	0.02(0.01–1.32)	ns	0.02(0.01–0.70)	**0.032**
2 *(*vs. *stage 4)*	0.08(0.01–0.49)	**0.007**	0.01(0.01–0.12)	**0.007**	0.01(0.01–0.07)	**0.004**
1 *(*vs. *stage 4)*	0.08(0.01–0.49)	**0.006**	0.01(0.01–0.62)	**0.027**	0.01(0.01–0.37)	**0.011**
**miR-3907** *(above* vs. *below median)*	2.37(1.66–3.40)	**<0.001**	3.21(1.63–6.32)	**0.001**	3.06(1.65–5.68)	**<0.001**

Bold values indicate the significant values (*p*<0.05)

## Discussion

The present study was undertaken in order to investigate the potential use of circulating miRs as biomarkers of the future clinical course of ADPKD patients. Autosomal dominant polycystic kidney disease (ADPKD) is the most common genetic cause of chronic kidney disease [1, 13]. While the disease mechanisms remain unclear, both the causal mutation, renal volume and the amount of hypertension are considered surrogate markers of progression while controlling hypertension is today the only widely accepted treatment [1, 13].

We found significant differing levels of multiple miRs both between ADPKD patients and healthy controls, as well as between ADPKD hypertensives (HT+) and non-hypertensives (HT-). In each case the differences were at most 3-fold, while the overlap between HT+ and HT- groups was significant and apparently not associated with blood pressure (Fig. 3a). Differences between CKD stages tended to be higher (up to 7-fold; Fig. 3b) and more systematic (Figs. 3, 4). Thus, our data suggests that as with most other biomarkers, renal function is an important consideration when using circulating miRs as biomarkers. It is nonetheless encouraging that at least one miR (miR-3907) showed a robust association with progression of eGFR decline in the patient group (Fig. 5). Copy numbers of miR-3907 were 2.7-fold higher in ADPKD patients than in healthy controls, with a 1.2-fold increase in ADPKD patients with hypertension as compared to ADPKD patients without hypertension. In addition, ADPKD patients with high copy numbers of miR-3907 were also more likely to lose >10% of their residual GFR during the one year follow-up.

While miR-3907 is not predicted to target mRNA derived from PKD1 or 2, the canonical ADPKD-associated genes, it is highly likely to bind to mRNA of the inositol 1,4,5-trisphosphate receptor (IP3R) interacting protein (IP(3)RIP) [14]. This is of interest as the receptor target of IP(3)RIP, called IP(3)R, is an intracellular Ca^2+^-channel in the endoplasmic reticulum reported to be a main effector of PKD 1 and 2-induced calcium signaling [15]. Indeed, the PKD 2-IP(3)R interaction may be instrumental for the formation of the microdomains necessary for IP(3)R Ca^2+^-amplified Ca^2+^ release. Similarly, IP(3)RIP has been demonstrated to robustly enhance Ca^2+^-mediated inhibition of IP(3)R Ca(2+) release [16]. Speculatively an impaired PKD-function would thus lead to lower IP(3)R Ca^2+^-release, triggering a compensatory downregulation of IP(3)RIP through miR-targeting of its’ mRNA.

The source cells and targets of the miRs identified in the present study may also the molecular pathogenesis of ADPKD. miR silencing of target mRNAs is mediated by complementary sequences (miR recognition element) located in the 3′ untranslated region (UTR) of the mRNA matching approximately 2–8 nucleotides (the seed region) at the 5′ end of the miR. Thus, any given miR is likely to have more than one target mRNA. Despite this complexity, recent research has elucidated specific roles for individual miRs in kidney development and physiology [9, 10, 17]. In ADPKD rodent models miR-15a is down-regulated [8]. Moreover, the miR-17 ~ 92 cluster is reported to be highly expressed in kidneys from several mouse models of PKD while its overexpression in normal mice produces renal cysts in wild-type mice [8, 18]. Based on these data and increased urine levels of miR-13-3b, Ben-Dov et al. have proposed a role of miRs as biomarkers in ADPKD [5].

As shown in Fig. 1, the miRs with significant differences in circulating copy numbers between patients and controls in our study preferrentially involved certain pathways. Of these ErbB signaling is targeted by miR-126-5p and miR-27a-3p, and our data is in this regard consitent with earlier reports of ErbB as an important signal for cyst enlargement in ADPKD [19, 20]. Overexpression of ErbB4 in mice induced renal cysts [21], whereas deletion attenuated disease progression in the *Cpk* mouse model [22]. Finally Aguado-Fraile et al. have suggested that miR-27a-3p may be useful as a marker of acute kidney injury even in the absence of ADPKD [23].

Other signalling pathways regulated by the miRs significantly different between the groups in our data include apoptosis, where miR-92a-3p and miR-202-5p have both been reported to display phenotype-relavant protective roles in models of IgA [6]. Finally, miR-4516 isolated from urinary exosomes has been proposed as a urine biomarker of sodium re-uptake in the kidney [24]. Taken together, these findings suggest that the miRs identified in our data are not random noise but reflect an underlying biology of a significance yet to be determined.

A number of weaknesses in the present study must be acknowledged. The absence of a non-ADPKD CKD control group is a weakness, especially as our data suggests that eGFR is a significant determinant of several miRs. Also, the limited number of patients combined with the lack of ultrasonographically measured renal volumes also limits the interpretation of the data. Nonetheless our data are among the first measuring miRs in ADPKD and may serve as a foundation for future candidate-based studies.

## Conclusions

The present study suggests (a) that random blood-sampling in a clinical setting can yield meaningful patterns of miRs, (b) that these patterns vary between healthy subjects and those with kidney disease, and, finally, (c) that increased copy numbers of miR-3907 in the circulation predicts the future decline in eGFR among ADPKD patients followed for 12 months.

## Additional file

Additional file 1: List of miRNAs included in the Qiagen miScript miRNA PCR Array Human Serum & Plasma 384HC in Excel format. (XLSX 12.5 KB)

## Abbreviations

ADPKD: Adult polycystic kidney disease
ESRD: Eend-stage renal disease
eGFR: Estimated glomerular filtration rate from biochemical data
miRs: micro-RNAs
qPCR: Reverse transcription reaction and quantitative real time polymerase chain reaction
ROC: Reciever operating characteristics methodology
